# Adapting with Age: Perspectives on Caretaking in Older Adult Romantic Relationships

**DOI:** 10.1093/geroni/igaf122.2971

**Published:** 2025-12-31

**Authors:** Katelyn Vozza, Jillian Rants, Melinda Heinz, William Henninger

**Affiliations:** University of Northern Iowa, Cedar Falls, Iowa, United States; University of Northern Iowa, Cedar Falls, Iowa, United States; University of Northern Iowa, Cedar Falls, Iowa, United States; University of Northern Iowa, Cedar Falls, Iowa, United States

## Abstract

Establishing and maintaining quality romantic relationships is an important part of older adulthood. However, older adults experience unique challenges related to health as they explore potential relationships that add meaning to their lives. The growing likelihood of health conditions may change relationship dynamics and introduce a caretaking dynamic into their relationships. This study investigated the differences in how a caretaking role is perceived among single older adults and those in committed relationships, or pursuing new relationships. Interviews were conducted from a sample of 20 older adults aged 65 + (N = 10 single, N = 10 committed relationships) who completed a survey on dating and relationships. Interviews were transcribed verbatim and analyzed using Braun and Clarke thematic analysis approach. Participants’ experiences related to past and current relationships often included caretaking responsibilities of a partner. Findings suggested that older adults in long-term committed relationships adapted to the changing health and needs of their partner, stating, “It’s something I kind of wish I wouldn’t have to do, but there have been times where I’ve been ill and he’s taken care of me, so…fair play.” Single/widowed older adults, however, viewed caretaking as a significant concern, “It was so hard to be supportive of somebody with a disability, and I just don’t think I could do it again.” Women in particular may be hesitant towards new caregiving responsibilities in later life, suggesting that couples may want to proactively discuss these concerns while seeking out romantic partners, and negotiate attitudes of independence and compromise.

